# Enhanced ICOS Signaling Between Dendritic Cells and T Cells Characterizes the Immune Landscape of Human Cholangiocarcinoma

**DOI:** 10.1155/humu/9981470

**Published:** 2025-10-23

**Authors:** Meiying Zhu, Yuou Li, Xiaolong Tang, Xinjian Wan, Zunqiang Zhou

**Affiliations:** ^1^Digestive Endoscopic Center, Shanghai Sixth People's Hospital Affiliated to Shanghai Jiao Tong University School of Medicine, Shanghai, China; ^2^Infectious Diseases Department, Harbin Sixth Hospital, Harbin, Heilongjiang, China; ^3^Department of General Surgery, Shanghai Eighth People's Hospital, Shanghai, China; ^4^Department of Surgery, Shanghai Sixth People's Hospital Affiliated to Shanghai Jiao Tong University School of Medicine, Shanghai, China

**Keywords:** cholangiocarcinoma, ICOS signaling, ICOS–ICOSL axis, single-cell RNA sequencing, tumor microenvironment

## Abstract

Cholangiocarcinoma exhibits a complex tumor microenvironment, yet the cellular interactions governing its progression remain poorly understood. Here, through integrated analysis of two independent single-cell RNA sequencing datasets comprising both complete tissue and immune-focused profiling, we comprehensively mapped the cellular landscape and intercellular communication networks in human cholangiocarcinoma. Our analysis revealed significant remodeling of immune cell compositions and interaction patterns in the tumor microenvironment. Notably, we identified enhanced ICOS signaling between dendritic cells and T cells as a prominent feature of cholangiocarcinoma. Using CellChat analysis, we demonstrated that tumor-associated dendritic cells, particularly plasmacytoid DCs, exhibit stronger ICOS-mediated communication with T cells compared to their counterparts in normal tissues. Functional validation experiments confirmed that tumor-conditioned dendritic cells upregulate ICOSL expression and promote CD8+ T-cell activation through the ICOS–ICOSL axis, as evidenced by increased CD69 and CD25 expression. This activation was specifically abolished by ICOSL blockade, establishing the functional significance of this pathway. Our findings provide novel insights into tumor-immune interactions in cholangiocarcinoma and suggest the ICOS–ICOSL axis as a potential therapeutic target for immunotherapy.

## 1. Introduction

Cholangiocarcinoma (CCA) is a rare yet aggressive malignancy of the biliary epithelium, representing the second most common primary liver cancer after hepatocellular carcinoma [1–4]. It is classified into three anatomical subtypes: intrahepatic (iCCA), hilar (hCCA), and extrahepatic (eCCA), reflecting differences in histogenesis and clinical presentation [1–3, 5].

The tumor microenvironment (TME) of CCA plays a critical role in tumor progression through complex interactions between various cellular components and signaling pathways. Key contributors to this environment include cancer-associated fibroblasts (CAFs), which secrete protumorigenic factors such as TGF-*β* and stromal cell–derived factor-1, and tumor-associated macrophages, which promote angiogenesis and immune suppression [1, 6–8]. These interactions are often mediated by cytokines, extracellular vesicles, and growth factors, emphasizing the TME's role in driving CCA invasiveness and resistance to therapy [1, 9, 10].

The ICOS–ICOSL (inducible T-cell costimulator–ICOS ligand) signaling pathway plays a significant role in regulating immune responses in cancers [11, 12]. ICOS, a costimulatory receptor expressed predominantly on activated T cells, interacts with its ligand ICOSL on antigen-presenting cells to enhance T-cell activation, survival, and cytokine production. This signaling axis is crucial for balancing immune tolerance and antitumor immunity. Previous studies have highlighted the important role of the ICOS–ICOSL pathway in various cancers, including CCA [13, 14]. On the one hand, ICOS promotes the activation of effector T cells, such as CD4+ T-helper cells and CD8+ cytotoxic T cells, which are essential for antitumor immunity and tumor control. On the other hand, sustained ICOS signaling has been associated with T-cell exhaustion, particularly in the context of the chronic inflammatory TME, which can impair effective immune responses [15–17]. In CCA, a malignancy characterized by immune evasion and poor prognosis, ICOS–ICOSL interactions may be pivotal in modulating the immune landscape and influencing the infiltration and activity of immune cells within the TME.

In this study, we integrate two independent single-cell RNA sequencing (scRNA-seq) datasets to comprehensively map the cellular landscape and intercellular communication networks in CCA. Through this analysis, we aim to further investigate the role of ICOS signaling in mediating dendritic cell (DC)–T cell interactions within the TME of CCA.

## 2. Results

### 2.1. Single-Cell Transcriptomic Profiling Reveals the Cellular Landscape of CCA

**Figure 1:**
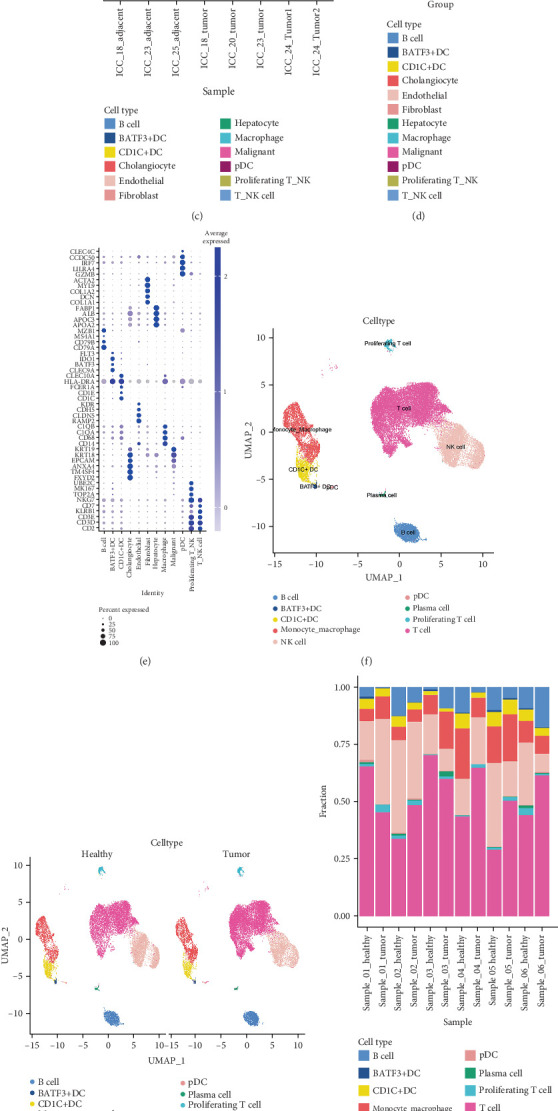
Cellular heterogeneity in cholangiocarcinoma revealed by dual single-cell RNA sequencing datasets. (a–e) Analysis of complete tissue dataset (GSE138709). (a) UMAP visualization of 12 major cell populations including malignant cells, immune cells, and stromal components. (b) Proportion of cell types between tumor and adjacent tissues. (c) Sample-level cellular composition showing heterogeneous distribution across individual samples. (d) Group-level comparison between tumor and adjacent tissues. (e) Heatmap showing expression of canonical marker genes validating cell type annotations. (f–j) Analysis of immune-focused dataset (GSE171899). (f) UMAP visualization of 10 distinct immune cell populations. (g) Cell type proportions between healthy and tumor conditions. (h) Sample-level analysis of immune cell distributions. (i) Group-level comparison showing consistent alterations in immune landscape between tumor and healthy tissues. (j) Expression patterns of canonical immune cell markers across identified populations.

Using scRNA-seq, we analyzed two independent datasets that provide complementary insights into the CCA TME. In the first comprehensive dataset (GSE138709), unsupervised clustering analysis revealed 12 major cell populations visualized on a uniform manifold approximation and projection (UMAP) plot (Figure 1). These populations included malignant cells, T/NK cells, B cells, various DC subsets (BATF3+ DCs, CD1C+ DCs, and pDCs), macrophages, endothelial cells, fibroblasts, cholangiocytes, and hepatocytes. Comparison of cellular compositions between tumor and adjacent tissues revealed significant alterations, particularly in the proportions of immune and malignant populations (Figure 1). Sample-level analysis demonstrated considerable heterogeneity in cellular composition across individual samples (Figure 1), while grouping samples by their tissue origin (tumor vs. adjacent) revealed consistent patterns of cellular alterations in the TME (Figure 1). To validate our cell type annotations, we examined the expression of canonical marker genes across all identified populations (Figure 1).

To further dissect the immune landscape, we analyzed a second dataset focused specifically on immune cells (GSE171899). This analysis revealed nine distinct immune cell populations, providing higher resolution of immune cell heterogeneity (Figure 1f); the identified immune populations are shown stratified by group (tumor vs. healthy) to visualize distributional differences (Figure 1g). The immune populations included T cells, NK cells, B cells, plasma cells, monocytes/macrophages, and three distinct DC subsets (BATF3+ DCs, CD1C+ DCs, and pDCs). Analysis of cellular composition at the individual sample level revealed sample-specific variations in immune cell distributions (Figure 1h), while comparison between tumor and healthy groups demonstrated consistent alterations in the immune landscape, particularly in the proportions of T cells and myeloid populations (Figure 1i). A subclustering analysis of T cells from the GSE138709 dataset was performed using Seurat and the Louvain algorithm (resolution = 0.1). Based on canonical marker genes, we identified distinct subsets, including CD8_activated, T_proliferating, CD4, and T_reg (see Figure S1A, B). UMAP and violin plots (Figure S1A, C) display the distribution of these subsets and ICOS expression levels, while the heatmap and KEGG pathway enrichment analysis (Figure S1B, D) highlight their functional diversity. Cell type identities were confirmed through examination of canonical immune cell markers, with distinctive expression patterns for lymphoid and myeloid populations (Figure 1j).

### 2.2. Rewired Intercellular Communication Networks in CCA

**Figure 2:**
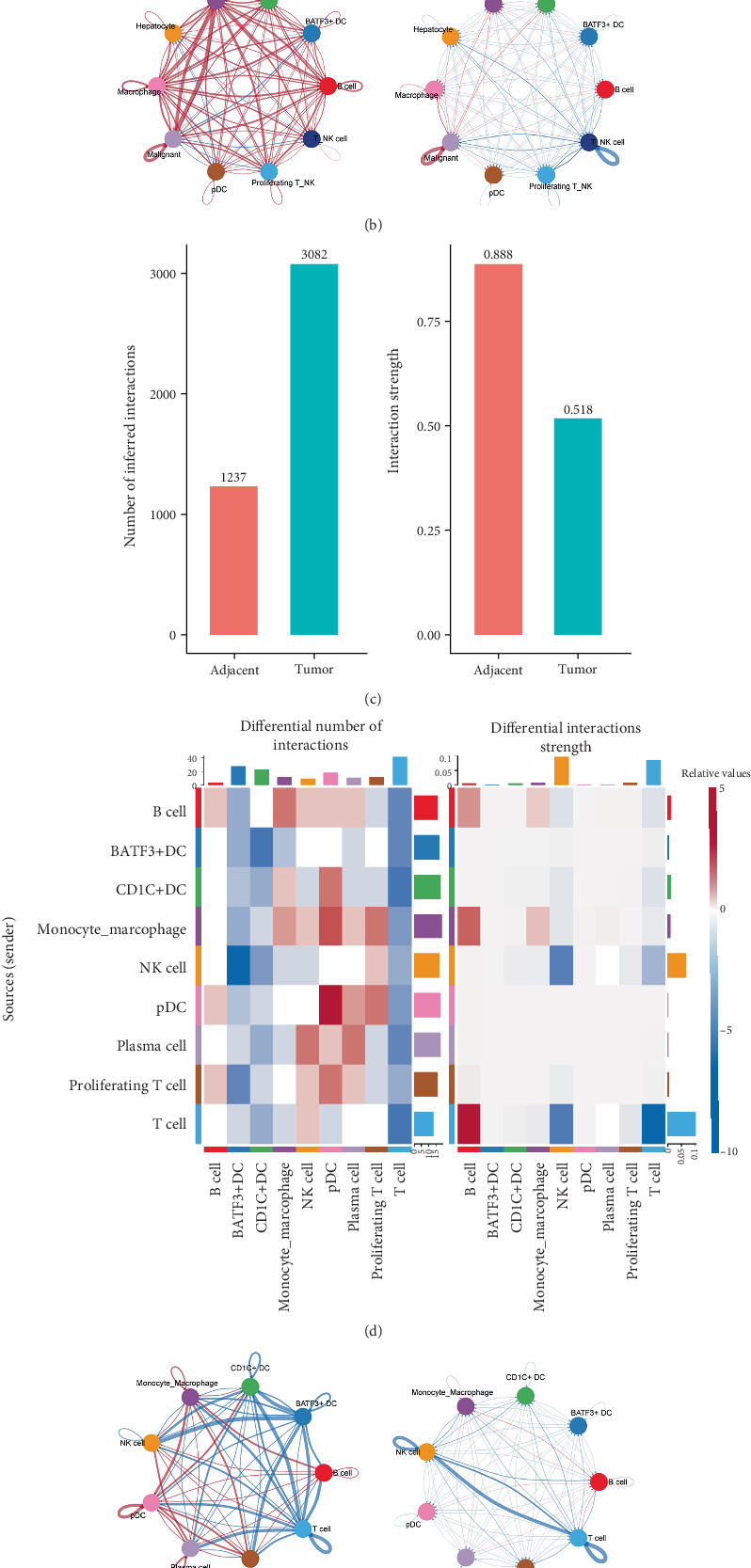
Rewired intercellular communication networks in cholangiocarcinoma. (a–c) Analysis of complete tissue dataset (GSE138709). (a) Differential number of interactions for each cell type as signal senders and receivers between tumor and adjacent tissues. (b) Relative interaction strength visualization highlighting qualitative changes in cellular communication. (c) Sender–receiver relationship analysis. (d–f) Analysis of immune-focused dataset (GSE171899). (d) Differential interaction patterns. (e) Relative interaction strengths. (f) Cell type–specific signaling patterns between tumor and healthy conditions.

To understand how cellular interactions are altered in the TME, we performed comprehensive cell–cell communication analysis using CellChat across both datasets. In the complete tissue dataset (GSE138709), we first quantified the differential number of interactions for each cell type as both signal senders and receivers between tumor and adjacent tissues (Figure 2a). This analysis revealed significant alterations in the communication network, particularly for malignant cells, macrophages, and T/NK cells. Examination of relative interaction strengths further demonstrated that these changes were not only quantitative but also qualitative, with certain cell types showing enhanced or diminished signaling intensities in the tumor context (Figure 2b). When examining the specific interaction patterns between different cell populations, we observed distinct sender–receiver relationships that were uniquely altered in the TME (Figure 2c).

Analysis of the immune-focused dataset (GSE171899) revealed more detailed changes in immune cell interactions. Quantification of differential interaction numbers showed marked changes between tumor and healthy conditions, with notable alterations in communication patterns among different immune cell populations (Figure 2d). The relative interaction strength analysis highlighted specific immune cell populations that displayed enhanced communication in the tumor environment (Figure 2e). Further investigation revealed cell type–specific patterns of incoming and outgoing signals that were distinctly different between tumor and healthy conditions (Figure 2f).

### 2.3. ICOS Signaling Network Exhibits Distinct Patterns in TME

**Figure 3:**
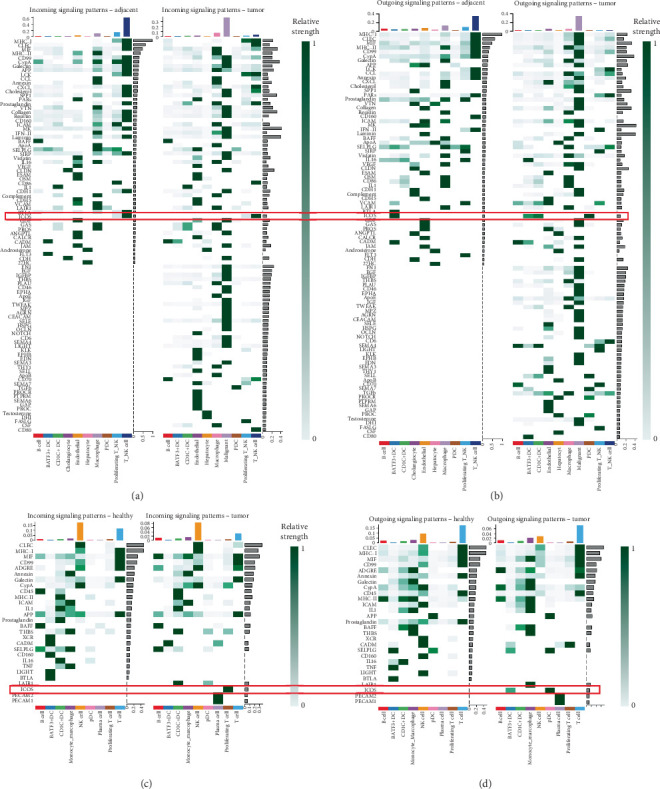
Global signaling pathway alterations in cholangiocarcinoma microenvironment. (a, b) Analysis of complete tissue dataset (GSE138709). (a) Incoming signaling patterns comparing adjacent versus tumor tissues. (b) Outgoing signaling patterns demonstrating pathway-specific alterations. (c, d) Analysis of immune-focused dataset (GSE171899). (c) Incoming signaling patterns in immune cells between healthy and tumor conditions. (d) Outgoing signaling patterns highlighting immune cell–specific pathway modulation in tumor environment.

**Figure 4:**
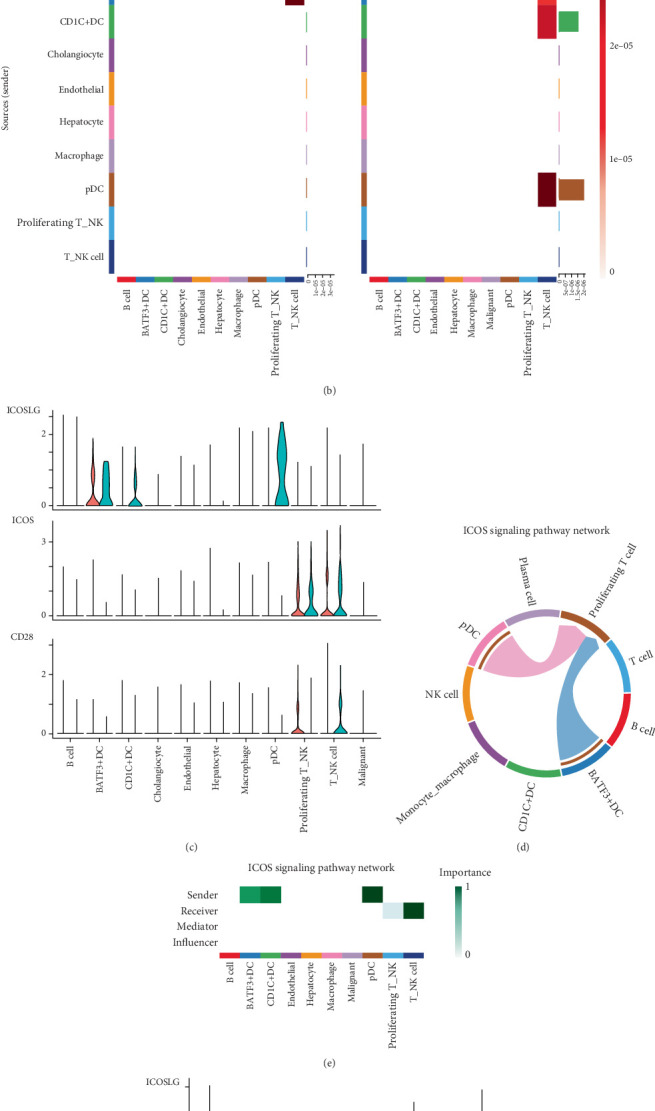
ICOS signaling network analysis reveals enhanced DC–T cell communication in the tumor microenvironment. (a–c) Analysis of the complete tissue dataset (GSE138709). (a) Circle plot visualization comparing ICOS signaling networks between adjacent and tumor tissues. (b) Quantitative analysis of ICOS signaling strength showing enhanced DC–T cell interactions. (c) Expression patterns of ICOS pathway components across cell types. (d–f) Analysis of the immune-focused dataset (GSE171899). (d) Immune cell–specific ICOS signaling network visualization. (e) Role analysis identifying key senders and receivers. (f) Detailed expression analysis of ICOS and ICOSL across immune cell populations.

To further characterize the enhanced ICOS signaling observed in CCA, we performed detailed pathway-specific analysis (Figure 3). In the complete tissue dataset (GSE138709), visualization of the ICOS signaling network revealed striking differences between adjacent and tumor tissues (Figure 4a). While adjacent tissue showed limited ICOS signaling primarily between T/NK cells and DCs, the tumor environment displayed a more complex network with additional interactions involving macrophages and other immune cells, indicated by increased connection lines between cell populations.

Quantitative analysis of sender–receiver relationships demonstrated significant changes in ICOS signaling strength and directionality (Figure 4b). In the adjacent tissue, BATF3+ DCs and CD1C+ DCs showed modest ICOS signaling to T cells. However, in the tumor condition, we observed notably enhanced ICOS signaling strength, particularly from pDCs to T/NK cells, as indicated by the darker colored bars representing stronger interaction intensity.

Expression analysis of ICOS signaling components revealed cell type–specific patterns (Figure 4c). ICOSL expression was predominantly observed in DC populations, with particularly high levels in tumor-associated pDCs. Correspondingly, ICOS expression was mainly detected in T-cell populations, while CD28, a related costimulatory molecule, showed a similar but distinct expression pattern. These expression patterns aligned with and supported the observed signaling network changes.

Analysis of the immune-focused dataset (GSE171899) provided further validation and additional insights into the ICOS signaling network. The circle plot visualization revealed a detailed immune cell communication network centered on ICOS signaling (Figure 4d), highlighting the primary roles of specific DC subsets and T cells in this pathway. The network strength analysis revealed distinct patterns of sender, receiver, mediator, and influencer roles among different immune populations (Figure 4e). Notably, pDCs and CD1C+ DCs emerged as key signal senders, while T cells served as the primary receivers, consistent with our observations from the complete tissue dataset.

Detailed expression analysis in the immune cell populations further supported these findings (Figure 4f). Expression patterns of ICOSL showed high specificity in DC subsets, particularly in BATF3+ DCs and pDCs, while ICOS expression was predominantly observed in T-cell populations. This complementary pattern of ligand–receptor expression reinforced the functional significance of DC–T cell ICOS signaling in the TME.

### 2.4. Enhanced ICOSL Expression in Tumor-Conditioned DCs Promotes CD8+ T-Cell Activation

**Figure 5:**
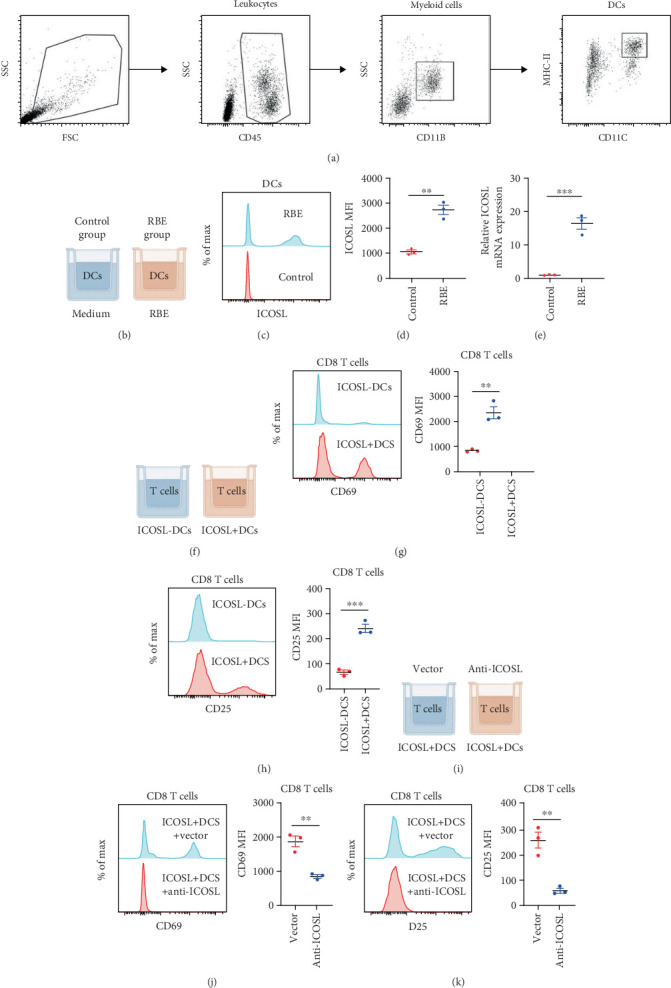
RBE induces the expression of ICOSL in DCs to enhance antigen presentation. (a) Strategies for DCs by flow cytometry. (b) Schematic diagram of DCs coculture with RBE cell line. (c, d) Representative flow cytometry results showed that RBE induced elevated expression of ICOSL on the DC surface. (e) RBE induces transcriptional enhancement of ICOSL in DCs. (f) Schematic diagram of DCs coculture with CD8 T cells. (g, h) ICOSL-DCs were unable to induce CD8 T-cell activation. (i–k) ICOSL blockade inhibited the activation of CD8 T cells by DCs. *n* = 3. Data are represented as mean ± SEM. ⁣^∗^*p* < 0.05;^∗∗^*p* < 0.01;^∗∗∗^*p* < 0.001;^∗∗∗∗^*p* < 0.0001.

To investigate the functional significance of ICOS signaling in CCA, we established an in vitro system to study DC–T cell interactions. We first isolated DCs from healthy donor PBMCs using a sequential gating strategy based on forward/side scatter characteristics, CD45 expression, CD11b positivity, and CD11c/MHC-II coexpression (Figure 5a). These purified DCs were then exposed to conditioned medium from the CCA cell line RBE or control medium in a Transwell coculture system (Figure 5b). Flow cytometric analysis revealed marked upregulation of ICOSL expression on DCs exposed to RBE-conditioned medium compared to control conditions (Figure 5c). This increase was consistently observed across multiple experiments, with significant elevation in both ICOSL protein levels (mean fluorescence intensity, MFI) (Figure 5d) and mRNA expression (Figure 5e).

To evaluate the functional consequences of enhanced ICOSL expression, we separated DCs into ICOSL+ and ICOSL− populations (Figure 5f) and assessed their ability to activate CD8+ T cells. Coculture experiments demonstrated that ICOSL+ DCs were significantly more effective at inducing T-cell activation, as evidenced by increased expression of the early activation marker CD69 (Figure 5g) and the IL-2 receptor alpha chain CD25 (Figure 5h) on CD8+ T cells. Quantification of these markers showed consistently higher expression levels in T cells exposed to ICOSL+ DCs compared to those cultured with ICOSL− DCs.

To confirm the specificity of ICOSL-mediated T-cell activation, we performed blocking experiments using anti-ICOSL monoclonal antibodies (Figure 5i). The enhanced T-cell activation observed with ICOSL+ DCs was significantly attenuated by ICOSL blockade, as demonstrated by reduced expression of both CD69 (Figure 5j) and CD25 (Figure 5k) on CD8+ T cells. These findings establish that tumor-induced ICOSL expression on DCs plays a crucial role in promoting CD8+ T-cell activation, suggesting that the enhanced ICOS signaling observed in CCA actively modulates antitumor immune responses.

## 3. Discussion

In this study, we integrated two independent scRNA-seq datasets to comprehensively analyze the cellular composition and intercellular communication networks within the TME of CCA. Our findings reveal significant remodeling of the immune landscape, particularly the enhanced ICOS–ICOSL signaling axis between DCs and T cells. Tumor-associated DCs, especially pDCs, showed upregulated ICOSL expression compared to normal tissue, facilitating stronger interactions with T cells and notably promoting CD8+ T-cell activation through the ICOS–ICOSL pathway. Additionally, substantial alterations in immune cell interactions and communication intensities within the TME suggest immune-related manipulation in tumor progression. Functional validation experiments confirmed the crucial role of ICOSL in mediating DC–T cell interactions under tumor-conditioned conditions, highlighting the potential of targeting the ICOS–ICOSL axis as a therapeutic strategy in CCA.

ICOS, a key member of the CD28 family, is expressed on activated T cells but can also be induced on other leukocyte subsets under specific conditions. It plays an essential role in immune modulation through its interaction with ICOSL on antigen-presenting cells, including macrophages, DCs, and B cells [18–20]. The ICOS signaling pathway enhances T-cell receptor (TCR)–mediated activation and promotes the proliferation and differentiation of effector T cells. ICOS is particularly crucial for follicular helper T cells (Tfh), which interact with B cells to support germinal center formation and high-affinity antibody production. For instance, in nasopharyngeal carcinoma, chemotherapy-induced activation of the STING pathway enhances major histocompatibility complex Class I expression and drives innate-like B-cell (ILB) activation. ILBs then utilize the ICOS–ICOSL axis to expand Tfh and Th1 cells, leading to enhanced cytotoxic T-cell activity within tertiary lymphoid structures and improved clinical outcomes [21]. In combination with CTLA-4 blockade, ICOS costimulation has been shown to remodel the immune milieu, enriching effector CD8+ T cells, Th1 CD4+ T cells, and M1-like proinflammatory macrophages. This synergy creates a feedback loop where interferon-gamma (IFN-*γ*) produced by effector T cells polarizes macrophages toward an antitumor phenotype, thereby sustaining T-cell infiltration and functionality [15]. Furthermore, ICOS regulates the immunosuppressive functions of regulatory T cells (Tregs), maintaining immune tolerance and homeostasis [17, 22, 23]. Notably, ICOSL has been implicated in tumor progression through interactions with ligands such as osteopontin, promoting metastasis via angiogenesis and enhanced migratory capacity [24]. ICOS is also predominantly expressed on tumor-associated Tregs in several cancers, including breast cancer and melanoma, while ICOSL is found on pDCs in cancers like breast, ovarian, and gastric cancers [25–29]. The interaction between pDCs and Tregs via ICOS–ICOSL signaling supports Treg expansion and survival, enhancing their immunosuppressive function through the activation of nuclear factor of activated T cells [30]. This dual functionality highlights the complexity of ICOS–ICOSL signaling in the TME and underscores its potential as a therapeutic target to enhance antitumor immunity while mitigating immunosuppressive and prometastatic effects.

In the context of CCA, our study identified enhanced ICOS signaling between DCs and T cells as a prominent feature of the TME, consistent with previous findings [18]. We further confirmed that ICOS signaling is essential in DC-mediated CD8+ T-cell activation, suggesting that this pathway may serve as a compensatory mechanism to maintain antitumor immunity in CCA. However, the ultimate outcome—whether it promotes antitumor immunity or induces immunosuppression through T-cell exhaustion—likely depends on the broader immunological context within the TME, which warrants further investigation.

Although this study provides important insights into the role of the ICOS–ICOSL axis in CCA, several limitations must be considered. First, the relatively small sample size of the scRNA-seq datasets may limit the generalizability of these findings. Furthermore, while ICOS signaling enhances CD8+ T-cell activation via DCs, its dual role in promoting both antitumor immunity and immunosuppressive effects through T-cell exhaustion remains poorly understood. The conditions under which ICOS signaling shifts toward protumor or antitumor responses need to be further elucidated through more detailed mechanistic studies.

In conclusion, this study highlights the critical role of the ICOS–ICOSL axis in shaping the immune landscape of CCA by enhancing DC-mediated activation of CD8+ T cells. The upregulation of ICOSL on tumor-associated DCs and its ability to modulate T-cell activity position this pathway as a promising therapeutic target in CCA. Targeting ICOS–ICOSL interactions could provide a novel approach to enhance antitumor immunity, potentially improving clinical outcomes in patients with CCA.

## 4. Methods

### 4.1. scRNA-Seq Data Analysis

We analyzed two independent scRNA-seq datasets: a complete tissue dataset (GSE138709) containing both tumor and adjacent samples and an immune-focused dataset (GSE171899) enriched for CD45+ cells. Raw gene expression matrices were processed using Seurat V4.0. Cells with fewer than 200 genes, more than 20% mitochondrial reads, or more than 6000 genes were excluded. Gene expression values were normalized using SCTransform, and variable features were identified using the “vst” method. To mitigate technical noise within each dataset, SCTransform was used to regress out the effects of sequencing depth, serving as a batch correction step. As the two datasets represent distinct biological contexts, they were processed independently without cross-dataset integration. Principal component analysis was performed using the top 2000 variable genes, and UMAP was used for dimensionality reduction. Cell clusters were identified using the Louvain algorithm with a resolution of 0.8.

### 4.2. Cell Type Annotation and Differential Expression Analysis

Cell types were annotated based on the expression of canonical marker genes. Differential expression analysis between groups was performed using the Wilcoxon rank-sum test. For visualization of marker gene expression, we used normalized and scaled expression values. Cell type proportions were calculated for each sample and compared between groups using Fisher's exact test.

### 4.3. Cell–Cell Communication Analysis

Intercellular communication networks were analyzed using CellChat V1.1. Expression data were normalized, and cell–cell interactions were inferred based on known ligand–receptor pairs. Differential interaction analysis between conditions was performed using the built-in statistical framework of CellChat. Interaction strengths were calculated based on the expression levels of ligand–receptor pairs and the abundance of interacting cell types. For pathway-specific analysis, we focused on significantly differential pathways (false discovery rate < 0.05). In the circle plot visualizations, line thickness represents the strength of cell–cell interactions, and different colors correspond to distinct cell types.

### 4.4. Blood Sample Collection

Peripheral blood was collected from 20 healthy volunteers. Blood was drawn into EDTA vacuum tubes and processed at room temperature within 1 h of collection. Trypan blue staining of PBMC was performed using SepMate-50 centrifuge tubes (STEMCELL Technologies Inc.) according to the manufacturer's protocol to quantify the number of viable cells. The viability of PBMC cells obtained was greater than 80%. Isolated PBMCs were subsequently cryopreserved in freezing solution (90% FBS with 10% DMSO). PBMC tubules were placed in Mr. Frosty (Thermo Fisher, Waltham, Massachusetts, United States) and stored at −80°C for 48 h before transferring to liquid nitrogen for long-term storage. To minimize batch effects, samples were processed together on the same day, thawed in a 37°C water bath, and centrifuged at 400 × g for 5 min, and the pellet was resuspended in cold staining buffer.

### 4.5. Cell Culture

Human hepatobiliary carcinoma cell line RBE was purchased from BNCC Biosciences (Beijing, China). Cells were cultured in complete DMEM (supplemented with 10% FBS [10099141C, Invitrogen, United States] and penicillin^−^streptomycin–glutamine mixture [10378016, Invitrogen, United States]) with 5% CO_2_ at 37°C. The medium was renewed every 3 days.

### 4.6. Drug Treatment

ICOSL/B7-H2/CD275 monoclonal antibody (67003-1-Ig) was purchased from Proteintech and was used at a concentration of 1 *μ*L/mL to block the function of ICOSL on the DC surface.

### 4.7. Flow Cytometry

The obtained PBMCs were resuspended in the antibody cocktail and incubated at room temperature for 1 h. After centrifugation at 1000 rpm for 5 min, the stained cells were resuspended in pre-cold 250 *μ*L PBS. Details of the specific antibodies used for flow cytometry were as follows (Table 1).

## Figures and Tables

**Table 1:** Flow cytometry antibodies.

**Antibodies**	**Clone type**	**Source**	**Application**	**Dilution**
CD45 monoclonal antibody (HI30), Brilliant Violet 421, eBioscience	HI30	Invitrogen	FC	1:1000
CD11b monoclonal antibody (ICRF44), PE, eBioscience	ICRF44	Invitrogen	FC	1:1000
CD11c monoclonal antibody (3.9), FITC, eBioscience	3.9	Invitrogen	FC	1:1000
HLA-DR/DP monoclonal antibody (MEM-136), APC	MEM-136	Invitrogen	FC	1:1000
CD275 (B7-H2) monoclonal antibody (MIH12), APC, eBioscience	MIH12	Invitrogen	FC	1:1000
CD69 monoclonal antibody (FN50), APC, eBioscience	FN50	Invitrogen	FC	1:1000
CD25 monoclonal antibody (CD25-4E3), FITC, eBioscience	CD25-4E3	Invitrogen	FC	1:1000

## Data Availability

The data that support the findings of this study are available from the corresponding authors upon reasonable request.

## Supporting Information

Supporting Information Additional supporting information can be found online in the Supporting Information section. Figure S1: Subclustering analysis of T cells. (A) UMAP showing T-cell subcluster distribution. (B) Heatmap of key marker gene expression across T-cell subsets. (C) Violin plot illustrating ICOS expression in each T-cell subset. (D) KEGG enrichment analyses indicating functional characteristics of each T-cell subset.
