# Supplementary figures and images for: Enhanced ICOS Signaling Between Dendritic Cells and T Cells Characterizes the Immune Landscape of Human Cholangiocarcinoma
Source: Hum Mutat. 2025 Oct 23;2025:9981470. doi: 10.1155/humu/9981470 (PMC12575039; doi:10.1155/humu/9981470)

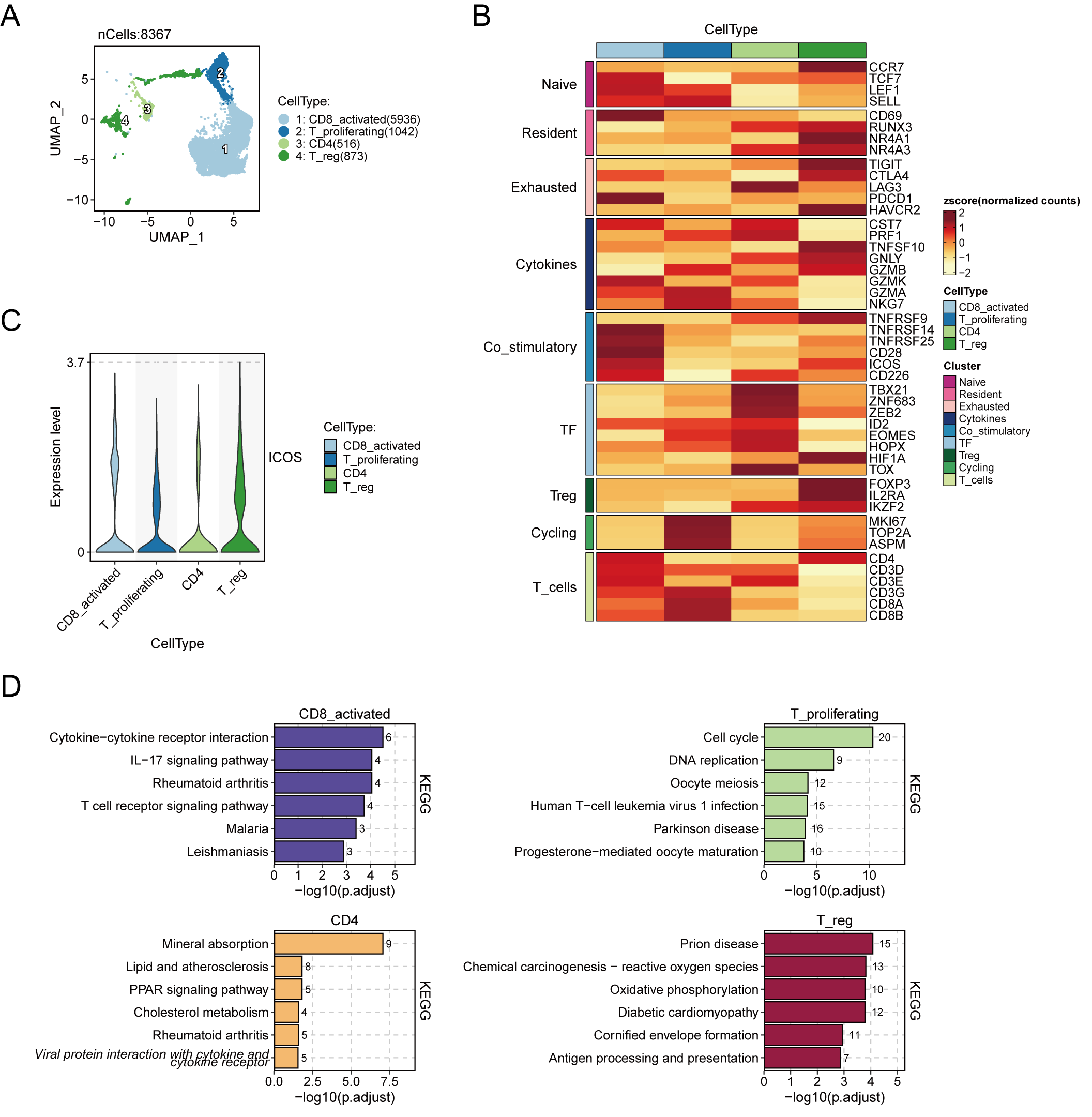

Supplement: Supporting Information — Additional supporting information can be found online in the Supporting Information section. Figure S1: Subclustering analysis of T cells. (A) UMAP showing T-cell subcluster distribution. (B) Heatmap of key marker gene expression across T-cell subsets. (C) Violin plot illustrating ICOS expression in each T-cell subset. (D) KEGG enrichment analyses indicating functional characteristics of each T-cell subset. [file 9981470.f1.tif]
